# Mix and manage: Cultivar mixtures can maintain yield under high wheat blast disease pressure

**DOI:** 10.1016/j.cropro.2024.106831

**Published:** 2024-10

**Authors:** Timothy J. Krupnik, Md. Harun-Or-Rashid, Dinabandhu Pandit, Rabiul Islam, Md. Khaled Hossain, José Mauricio Cunha Fernandes, Krishna Kanta Roy, Muhammad Rezaul Kabir, Sabine Stuerz, Pawan Kumar Singh, Golam Faruq

**Affiliations:** a International Maize and Wheat Improvement Center (CIMMYT), Dhaka, Bangladesh; b Independent Consultant, Dhaka, Bangladesh; c Bangladesh Wheat and Maize Research Institute (BWMRI), Dinajpur, Bangladesh; d Empresa Brasileira de Pesquisa Agropecuária (EMBRAPA) Trigo, Brazil; e Independent Consultant, Stuttgart, Germany; f International Maize and Wheat Improvement Center (CIMMYT), El Batan, Mexico

**Keywords:** Wheat blast resistance, Cultivar mixtures, Disease-inflicted yield loss, *Magnaporthe oryzae* pathotype *Triticum*, *Bipolaris* leaf blight

## Abstract

Originating in South America, wheat blast disease has spread to both Asia and Africa and is considered a significant threat to food security. Bangladesh experienced the first outbreak of wheat blast outside of the Americas in 2016. Shortly thereafter, the blast-resistant variety BARI Gom 33 was released. Seeds of this variety are however not as widely available as required, although the disease threat remains. While varietal mixtures have been shown to mitigate some symptoms and yield losses associated with other fungal diseases in wheat, there is a complete research gap on this topic as it pertains to wheat blast. As such, we evaluated the potential of using BARI Gom 33 as a component of a variety mixture under high disease pressure in Bangladesh. During three cropping seasons, blast symptoms and yield were determined in a field experiment for the highly blast-susceptible variety BARI Gom 26, the moderately susceptible BARI Gom 30, the resistant BARI Gom 33, and seven mixture combinations of the three varieties using artificial inoculation to increase disease pressure. In addition to wheat blast, *Bipolaris* leaf blight (BpLB) symptoms were observed and evaluated. While yields of the susceptible varieties were severely affected by blast even after fungicide application, disease-inflicted yield loss without fungicide was only 15% for sole BARI Gom 33 and did not differ significantly from yield losses in BARI Gom 33 and BARI Gom 30 mixtures. Furthermore, in the mixture containing 67% BARI Gom 33 and 33% BARI Gom 30, blast incidence and severity were reduced by 25% and 16%, respectively, in comparison to weighted values in sole stands. Conversely, mixing varieties tended to increase the symptoms of BpLB. Under high wheat blast pressure, fungicide protection against blast was relatively weak, underscoring the importance of resistant varieties. Although variety mixtures did not increase yield, the yield advantage of BARI Gom 33 was maintained when its seeds were mixed with the less resistant BARI Gom 30. This study confirms recommendations that farmers should use BARI Gom 33 as a first line of defense against wheat blast in Bangladesh. Yet where farmers cannot access sufficient BARI Gom 33 seed for planting, our data suggest that agricultural extension services can recommend this variety with non-resistant cultivars as interim strategy without significant risk of yield loss.

## Highlights

• Wheat blast globally threatens, expanding in Bangladesh despite the release of blast-resistant BARI Gom 33.
• Limited availability of resistant variety led to a study mixing it with susceptible varieties under high disease pressure.
• Mixing BARI Gom 30 with BARI Gom 33 seeds showed promising loss mitigation.
• Fungicide controlled BpLB effectively but struggled against blast.
• Using BARI Gom 33 in mixtures addresses wheat blast amid seed challenges in Bangladesh.

## 1 Introduction

Originating in South America, wheat blast is caused by the ascomycetous fungus *Magnaporthe oryzae* pathotype *Triticum* (*MoT*) (syn. *Pyricularia oryzae*). It is a significant disease that threatens over 6.4 million hectares of wheat globally (Pequeno et al., 2024). Originating in Brazil in 1985, the disease initially remained limited to South America until February of 2016 when 15,000 ha across eight districts of Bangladesh were suddenly affected (Islam et al., 2016). Although the outbreak resulted in the loss of only about 3% of the country's wheat production (Sadat and Choi, 2017), estimates are that farmers nonetheless lost up to $2.1 million in foregone profits (Mottaleb et al., 2023). Because this was the first occurrence of wheat blast outside South America (Malaker et al., 2016), concerns of possible spread to other wheat-producing countries increased following the 2016 outbreak (Chowdhury et al., 2017), and in 2018 the disease was identified in Zambia (Pequeno et al., 2024).

Wheat blast can cause severe yield losses of more than 80% (de Coelho et al., 2016). It is mainly a head disease (Kohli et al., 2011), with the most visible symptom being the bleaching of the spike. Infection of the rachis or peduncle damages the spike leading to partial or total sterility (Cruz and Valent, 2017). Wheat blast development is facilitated by high temperatures and high air humidity especially at time of heading (Montes et al., 2022). The 2016 outbreak in Bangladesh was also likely favored by unusually warm and humid weather in affected districts (Islam et al., 2019). To limit the spread of the disease by infected seeds to other countries, research has suggested that trade of seeds from blast endemic areas must be strictly regulated (Ceresini et al., 2018). In addition, fungicide application and the use of resistant varieties are the methods of choice to control blast in endemic areas. However, since fungicides do not always provide effective protection under high blast pressure (Zhang et al., 2022), host resistance is usually preferred (Randhawa et al., 2019). In 2017, the blast-resistant variety BARI Gom 33 (hereafter BARI Gom 33) was released in Bangladesh (Mottaleb et al., 2019) in response to these concerns, and as a partial solution to wheat blast (Hossain et al., 2019).

Another fungal disease of concern in Bangladesh is *Bipolaris* leaf blight (BpLB), or spot blotch, which is caused by *Bipolaris sorokiniana* (Sacc.). This pathogen causes both foliar and root diseases (Figueroa et al., 2018). Before the first outbreak of wheat blast in Bangladesh, BpLB was described as among the most destructive wheat fungal pathogens in tropical and sub-tropical climates (Acharya et al., 2011; Siddique et al., 2006). Early symptoms of BpLB are small, dark brown lesions, and the disease has caused significant yield losses especially in the North-Eastern Plains Zone (NEPZ) of India, the lowland Terai of Nepal, and in northwestern Bangladesh (Chowdhury et al., 2013).

Wheat seed supply in South Asia is almost entirely dependent on government agencies, although supplies typically fall short of demand (Jaim and Akter, 2019). The remaining seed demand is met by farmers from their own seed stock or from local seed dealers (Sadat and Choi, 2017). With the emergence of wheat blast, providing farmers with resistant varieties is a key objective, although it can take many years before seed multiplication and dissemination programs can affect large-scale adoption of new varieties. One way to mitigate this problem could be the use of seed mixtures, as farmers could mix in seeds from their own stock if there is insufficient supply of resistant variety. Varietal mixtures have been found to stabilize yields and mitigate disease-related yield losses due to barrier and frequency effects in several agroecosystems (Finckh et al., 2000; Vestergaard and Jørgensen, 2024). They have also been successfully used to control other fungal diseases in wheat such as stripe rust (Huang et al., 2012), Cephalosporium stripe (Mundt, 2002), and leaf rust (Jackson and Wennig, 1997).

BARI Gom 33 was developed by the Bangladesh Agricultural Research Institute (BARI) and released in 2017. It has gained popularity for its yield potential, adaptability, and disease resistance. This variety was derived from the cross between Kachu and Solala, carrying the *Aegilops ventricosa* 2N^v^S translocation segment conferring blast resistance. BARI Gom 33, as reported by BARI (BWMRI 2019), demonstrated a yield advantage of 5–8% compared to commonly grown wheat varieties. This variety is also recognized for its high zinc content, offering nutritional advantages. Because a resistant variety can serve as a barrier and as a pathogen dilutant in a mixed variety stand (Garrett and Mundt, 1999), mixing the resistant BARI Gom 33 with a more susceptible variety could also result in reduced blast incidence and severity in the crop stand. Following harvest, different wheat seed varieties are commonly mixed before milling in Bangladesh (Krupnik et al., 2022). This suggests that seed varietal mixtures could be used without compromising farmer's ability to produce wheat-based products or sell them into the market.

In response to these issues, the objectives of this study were (1) to evaluate the potential of wheat cultivar mixtures to mitigate wheat blast and BpLB compared to sole varietal stands, (2) to determine disease-inflicted yield loss based on yield with and without fungicide application, and (3) to discuss the scope for using BARI Gom 33 as a component variety on a larger scale.

## 2 Materials and methods

### 2.1 Experimental design and crop management

**Fig. 1:**
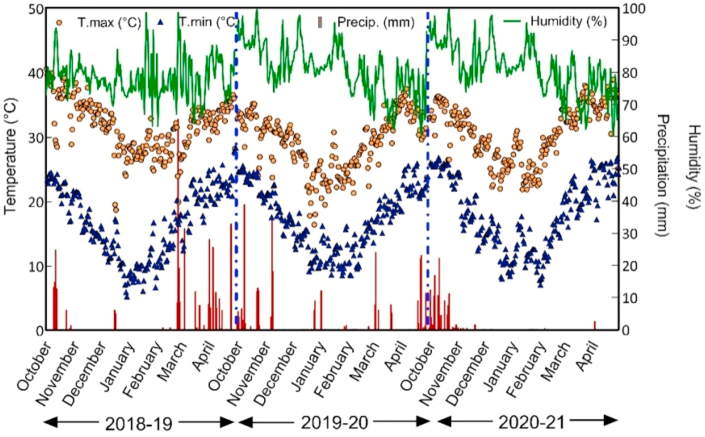
Temperature, precipitation and relative humidity measured during the wheat growing seasons from October to April of 2018–2019, 2019–2020, and 2020–2021 measured at the experimental location in Jashore, Bangladesh.

The experiment was conducted over three growing seasons between December 2018 and April 2021 in Jashore, Bangladesh (23°18′83.8″N, 89°19′00.3″E). Wheat was cultivated in ten variety mixtures during three cropping seasons, with sowings on December 23, 2018, December 23, 2019 and December 25, 2020. The variety mixtures contained different proportions of three wheat genotypes including (1) BARI Gom 26, a highly blast-susceptible non-2NS yet widely adopted variety (BARI 2017a), (2) BARI Gom 30, a moderately blast tolerant but non-2NS cultivar (BARI 2017b) and (3) BARI Gom 33, a 2NS blast-resistant cultivar (BWMRI 2019). Before sowing, seeds in the mixtures were thoroughly mixed according to official seeding rates advised by national research institutes (BARI 2017b; BWMRI 2019) at 120 kg ha^−1^ (Table 1). Weather data collected from an automatic weather station located at the experimental site is shown in Fig. 1.

**Table 1:** Mixtures used in the experiment with the respective proportions of each variety in percent plant density.

Mixture	Percent of each variety mixed		
	BARI Gom 26	BARI Gom 30	BARI Gom 33
BARI Gom 26(100)^a^	100	0	0
BARI Gom 30(100)^b^	0	100	0
BARI Gom 33(100)^c^	0	0	100
BARI Gom 26(67) + BARI Gom 30(33)	67	33	0
BARI Gom 26(67) + BARI Gom 33(33)	67	0	33
BARI Gom 26(33) + BARI Gom 30(67)	33	67	0
BARI Gom 26(33) + BARI Gom 33(67)	33	0	67
BARI Gom 30(67) + BARI Gom 33(33)	0	67	33
BARI Gom 30(33) + BARI Gom 33(67)	0	33	67
BARI Gom 26(33) + BARI Gom 30(33) + BARI Gom 33(33)	33	33	33

a Released 2010. Crosses: ICTAL 123/3/RAWAL87//VEE/HD 2285. BARI Gom 26 is a shorter height, medium tillering, tolerant to BpLB, mid duration variety (BARI 2017a; Gade et al., 2021).

b Released 2014. Crosses BAW 677/Bijoy. BARI Gom 30 is a short stature, early to mid-duration, moderately tolerant to BpLB variety (BARI 2017b; Gade et al., 2021).

c Released 2017. Crosses KACHU/SOLALA. BARI Gom 33 carries the 2NS translocation conferring blast resistance and is a Zn enriched, moderately susceptible to BpLB. tall, low-to-moderate tillering, and slightly late maturing variety (BWMRI 2019; Gade et al., 2021).

The experiment was laid out in a fully randomized design in five replications, with mixtures as treatments and fungicide applied and not applied to each of the mixtures described in Table 1. A 0.9 m spacing was maintained between all plots. The ten mixtures were randomly distributed with sole stands and mixtures sown in 1.8 m long plots with 10 rows at 0.2 m spacing between rows.

All agronomic practices were applied uniformly across treatments. Following three tillage passes of a two-wheel tractor drawn power tiller, urea (150 kg ha^−1^), triple superphosphate (135 kg ha^−1^), potassium chloride (110 kg ha^−1^), gypsum (110 kg ha^−1^) and boric acid (6.5 kg ha^−1^) were applied basally. An additional 75 kg ha^−1^ urea was applied at crown root initiation. Weed control was completed twice by hand, prior to fertilization at crown root initiation and eight weeks after sowing to maintain weed-free conditions. Plots were flood irrigated to 3–5 cm depth three days before sowing and 3–5 days before weeding corresponding with crown root initiation, and at heading. No pests or diseases other than wheat blast and BpLB were observed.

### 2.2 Disease inoculation

Each replicate was surrounded by three border rows of BARI Gom 26 located 1 m from main plot edges. These borders were inoculated every seven days with blast spores for six weeks, starting at the maximum tillering stage and ending at the milking stage. This corresponds to the period during wheat blast is most likely to cause infection and yield loss (Urashima et al., 2016). A protocol modified from Cruz et al. (2016) was used to produce and multiply inoculum of *MoT*. After being cultured on potato dextrose agar (PDA) for 10 days, mycelium plugs were transferred onto the oatmeal agar (OMA). The OMA was prepared using 50 g rolled oats and boiled in 500 ml distilled water at 70 °C for 1 h, filtered through 4 layers of grade 100 cheese cloth (56 × 41 threads inch^−1^) adjusted to 1 L with distilled water by adding 15 g agar powder and then autoclaved. 5 mm diameter PDA mycelial round blocks of *MoT* isolates were made using a sterile steel block puncher and transferred onto OMA Petri dishes. For rapid spore production, the agar plates were incubated under 26–28 °C and 40–50% RH in a clean bench chamber with 24 h fluorescence light.

After 10–12 days, sporulated plates were flooded with 5–10 ml distilled water and gently scrapped with camel hairbrush to harvest spores. Spores were collected in a 250 ml beaker and filtered through 2 layered cheese cloth. The spore suspension was gently stirred using a magnetic stirrer while adding a solution of 0.1% Tween 20 detergent. The suspension was subsequently adjusted to a concentration of ±40,000 spores ml^−1^ of water using a haemocytometer under a compound microscope.

To maintain a favorable environment for disease with the effective dispersal of the spores, all plots were mist irrigated. 150 mist ACU-Mister sprinklers (flow rates: 14.4 to 116.8 lph, working pressure: 0.7–2.5 kg/cm^2^, wetted radius: 1.5–2.9 m) from Jain Irrigation Systems Ltd. (Jalgaon, India) were installed and operated for 40 days daily from 11 a.m. to 5 p.m. for 10 min h^−1^ at the start of the hour, starting 2 days before the first inoculation at maximum tillering.

### 2.3 Disease management

Fungicide was both applied (+fungicide) and not applied (-fungicide) to each of the mixtures described in Table 1. Plots receiving fungicide were sprayed using a calibrated sprayer with Nativo 75WG (Bayer Crop Science; trifloxystrobin and tebuconazole AI) at 297 g AI ha^−1^ five days after each inoculation with blast spores as described below (+fungicide). To avoid contamination of non-treated plots, fungicides were sprayed when wind was at a minimum and with 2 m tall plastic sheeting placed in the center of alleys between plots and completely surrounding each plot sprayed. Sheeting was left in place for a minimum of 30 min after application to allow fungicide to settle on the canopy of the targeted plot before they were removed.

### 2.4 Disease and yield assessment

**Fig. 2:**
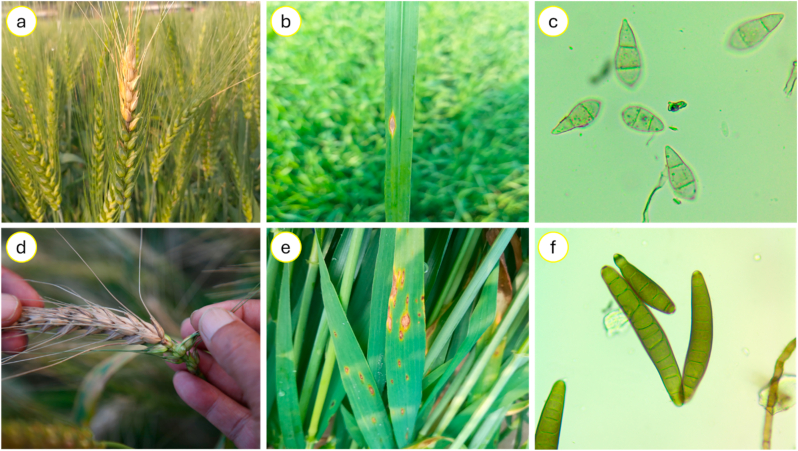
Wheat blast and *Bipolaris* leaf blight (BpLB) symptoms on different plant organs with microscopic views of each pathogen. (a) A typical wheat blast infected and partially ‘bleached’ spike observed in the trial. (b) A typical ‘eye-shaped’ leaf lesion with a gray or whitish center surrounded by dark brown margin. (c) A gray-colored two-septate pyriform hyaline to pale, asexual conidia of *MoT* under compound microscope (100x magnification). (d) A partially ‘bleached’ blast infected spike showing detail of the point of infection, (e) BpLB infected leaves with small brown lesions and elongation of necrotic tissue. (f) Olive-brown colored three to ten septate elliptical conidia of *Bipolaris sorokiniana* with tapered ends that are slightly curved, with smooth conidial walls with thickenings at the septa as observed under a compound microscope (100x magnification).

Excluding a border row of 20 cm from both ends, 30 plants were selected from 8 rows within 1.6 m × 1.4 m area at the varietal percentage of the corresponding mixture treatment plot to collect disease-related data. Blast incidence and severity of infected spikes, the primary plant organ affected by blast (Singh, 2017), and the percentage leaf area, the main organ affected by BpLB (Duveiller and García Altamirano, 2000), were visually scored independently for each plant by two trained experts three times during the season, with the average value observed by each expert recorded (Fig. 2). The first assessment was made at 75 Zadok's growth stage (ZGS) (Zadoks et al., 1974), about five weeks after the first inoculation and three weeks after heading (Table 2). The second and third assessments followed four and nine days after the first scoring, corresponding to 80 and 85 ZGS.

**Table 2:** Dates of sowing, average heading and maturity, and of 1st inoculation with blast spores and Zadok growth stages (ZGDs) during which disease assessment was conducted during the experiment.

	Year 1 (2018–19)	Year 2 (2019–20)	Year 3 (2020–21)
Sowing date	23.12.18	23.12.19	25.12.20
Mean heading date	19.2.19	18.2.20	21.2.21
1st inoculation	7.2.19	2.2.20	17.1.21
1st scoring (75 ZGS)	11.3.19	12.3.20	14.3.21
2nd scoring (80 ZGS)	15.3.19	16.3.20	18.3.21
3rd scoring (85 ZGS)	20.3.19	21.3.20	23.3.21
Mean maturity date	1.4.19	3.4.20	5.4.21

Lodging was visually assessed by two independent observers as the percentage of plants lodged per plot at maturity, with scores averaged for each plot. Yield was determined from the same central sub-plot area used for disease assessment on a surface of 2.24 m^2^. Grain moisture was determined with a Grain Moisture Meter (GMK-303RS, Korea) and grain yield was corrected to 12% moisture. Thousand grain weight (TGW) was calculated from the number of grains randomly sampled from harvested plants and corrected to 12% moisture content.

### 2.5 Computations

Wheat blast incidence was calculated as a percentage of all spikes of the selected plants in the area of observation (1.0 m × 2.1 m) following Equation (1).

(1) $$Wheat\;Blast\;incidence\;\left(\%\right)=\frac{{spikes\;no}_{\inf}}{{spikes\;no}_{tot}}\times 100$$

where *no* indicates the number and inf indicates infected spikes observed among the total number (*tot*) of plants sampled (*n* = 30).

Blast severity was computed as the average percentage of infected spikelets from 30 plants with Equation (2),

(2) $$Wheat\;Blast\;severity\;\left(\%\right)=\frac{\sum_{i=1}^{n}\frac{\;spikelets{\;no}_{\inf}}{spikelets{\;no}_{tot}}\times 100}{n}$$

where the number of blast infected spikelets and the total number of spikelets on each of the selected plants were counted and the percentage of infected spikelets calculated.

Disease index (DI) is the product of incidence and severity (Groth et al., 1999). It was calculated as the product of the proportion of infected spikes and the average proportion of infected spikelets (Equation (3)).

(3) $$Disease\;index\;\left(\%\right)=\frac{{spikes\;no}_{\inf}}{{spikes\;no}_{tot}}\times \frac{\sum_{i=1}^{n}\frac{{spikelets\;no}_{\inf}}{{spikelets\;no}_{tot}\;}\;}{n}\times 100$$

Flag leaves of each of the selected plants independently were visually assessed by the same two observers and averaged. The percentage of BpLB affected area of each of the selected plants was then calculated following Equation (4) as the average percentage of flag leaf area infected for the 30 sampled plants.

(4) $$BpLB\left(\%\right)=\frac{\sum_{i=1}^{n}\frac{{flag\;leaf\;area}_{\inf}}{{flag\;leaf\;area}_{tot}\;}\times 100}{n}$$

Because blast and BpLB infestations were also observed in the fungicide-treated plots, with the exception of BARI Gom 33, disease-inflicted yield losses were calculated using sole BARI Gom 33 treatments as a reference yielding ability of BARI Gom 30, BARI Gom 26, and their mixtures. The mean yield of BARI Gom 33 with fungicide in each year was therefore used as the reference (*reference*) yield for disease-free (*actual*) conditions in the assessment of disease-inflicted yield losses (Equation (5)).

(5) $$Disease-inflicted\;yield\;loss\;\left(\%\right)=\frac{{Yield}_{reference}-{Yield}_{actual}}{{Yield}_{reference}}\times 100$$

Differences in yield and disease-related parameters associated with growing plants in a mixture compared to pure stands have been described using the relative mixing effect (Kiær et al., 2009). The relative mixing effect was calculated as the relative advantage or disadvantage of actual measured values compared to expected values (Equation (6)), and was calculated as the weighted values of pure stands (Equation (7)).

(6) $$Relative\;mixing\;effect\;\left(\%\right)=\frac{{Value}_{measured}-{Value}_{expected}}{{Value}_{expected}}\times 100$$

(7) $$Expected\;value=a\times {Value}_{BG26}+b\times {Value}_{BG30\;}+c\times {Value}_{BG33}$$

where *a*, *b* and *c* are the proportion of the variety in the respective mixture. To rank the sensitivity of the mixtures to disease, the mean disease indices of the pure variety stands at 85 ZGS were used as the baseline for the sensitivity of each variety, with a sensitivity factor of the variety mixtures calculated as the weighted sensitivity using the DI of the pure stands (Equation (8)).

(8) $$Sensitivity\;factor=a\times {DI}_{BG26}+b\times {DI}_{BG30}+c\times {DI}_{BG33}$$

### 2.6 Statistical analysis

Before analysis, all data were tested for normal distribution using Shapiro-Wilk test at a significance level of *P* < 0.01. Non-normal distributions were observed for yield data only; accordingly, these data were transformed using the square root function prior to analysis. Because climatic conditions, which vary from season to season, can have a large effect on wheat blast (Islam et al., 2019), we determined the effects of year, mixture, and their interaction on yield, disease-inflicted yield loss, and relative mixing effect on yield, using 2-way analyses of variance (ANOVA). ANOVAs were performed separately for each mixture treatment that received fungicide or no fungicide. For the comparison of means, the Tukey-Kramer test was used at a significance level of *P* < 0.05. Because the data on relative mixing effects on disease-related parameters were not normally distributed and data transformation did not lead to satisfactory results, the non-parametric Kruskal-Wallis test was used to determine the effect of mixture. For comparison of means, the pairwise Wilcoxon test including Bonferroni correction was used. Relationships between disease related parameters, yield, TGW and lodging across years, fungicide and varietal treatments were explored using Pearson's correlation and linear regression. All analyses were performed using R (R core team, 2021).

## 3 Results

### 3.1 Yield and yield-related parameters

#### 3.1.1 Yield without fungicide

**Fig. 3:**
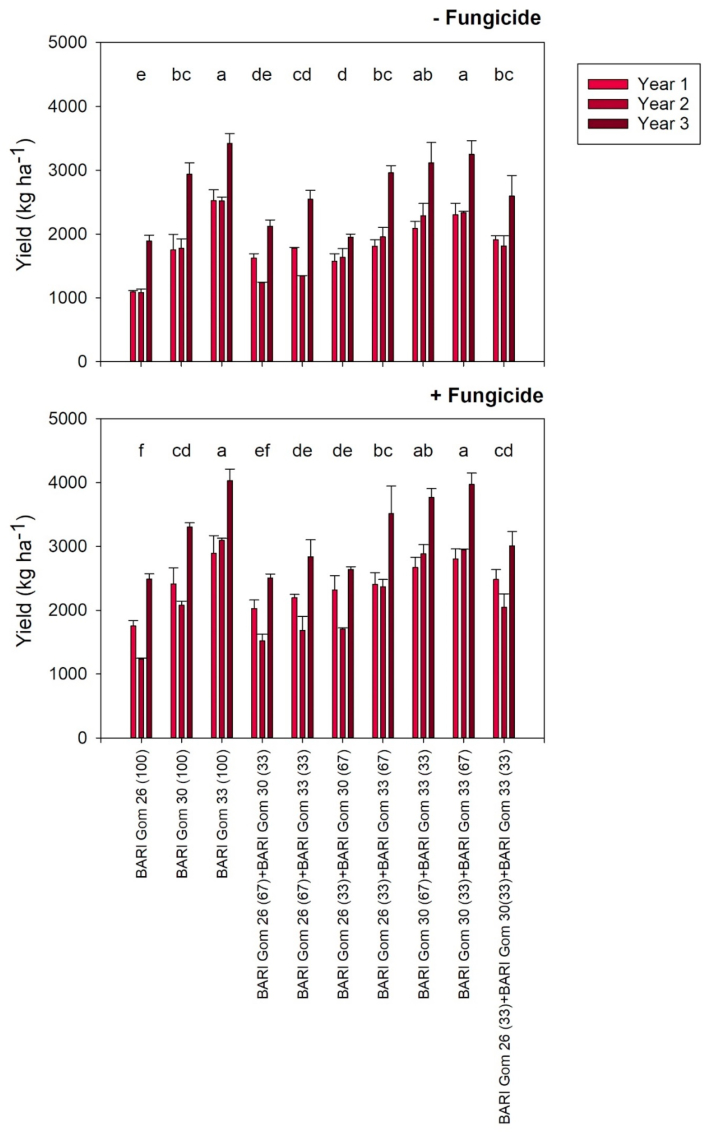
Moisture adjusted grain yield of three wheat varieties cultivated in pure variety stands and seven mixtures over three consecutive years. The experiment was conducted either with or without fungicide application. Mean values of 5 replications with standard errors are presented. Different letters indicate significant differences between mixtures across years at *P* ≤ 0.05. Letters that are not the same indicates statistical differences.

Without fungicide application and under high wheat blast disease pressure conditions, yield was significantly higher in Year 3 at 2679 kg ha^−1^ on average across mixtures, than in Year 1 with 1846 kg ha^−1^ and in Year 2 with 1800 kg ha^−1^ (Fig. 3). However, no significant interaction was found between the effects of year and mixture on yield (Table 3), and as such, mixtures were directly compared. The highest yield was obtained in the pure stand of the blast-resistant variety BARI Gom 33 with 2822 kg ha^−1^ on average across years. In addition, both mixtures of BARI Gom 33 with the moderately blast-tolerant variety BARI Gom 30 achieved statistically equivalent yields to the BARI Gom 33 pure stand at 2630 kg ha^−1^ (BARI Gom 30(33) + BARI Gom 33(67)) and 2497 kg ha^−1^ (BARI Gom 30(67) + BARI Gom 33(33)). The lowest yield was observed in the pure stand of the wheat blast-susceptible variety BARI Gom 26 with 1357 kg ha^−1^ on average across years. The mixtures containing 67% BARI Gom 26 also had relatively low yields, with the BARI Gom 26(67) + BARI Gom 30(33) mixture yielding 1662 kg ha^−1^, statistically indistinguishable from sole BARI Gom 26. Conversely, 1890 kg ha^−1^ was achieved with BARI Gom 26(67) + BARI Gom 33(33), slightly but significantly (*P* < 0.0001) higher than sole BARI Gom 26 across years. With 2155 kg ha^−1^, the yield of the sole stand of BARI Gom 30 was in the middle range and statistically comparable to the BARI Gom 26(33) + BARI Gom 30(67), BARI Gom 30(67) + BARI Gom 33(33) and the three-varietal mixtures (see Table 4).

**Table 3:** ANOVA of the effects of year, mixture and their interaction on yield without and with fungicide application.

	df	SS	MS	*F* value	*P* value
Without fungicide					
Year	2	7623121	1905228	115.7	<0.0001
Mixture	9	12306064	151944	32.7	<0.0001
Year x Mixture	18	97969	303	1.4	0.1180
Residuals	120	2050624	142		

With fungicide					
Year	2	8473921	2118480	105.9	<0.0001
Mixture	9	13461561	166219	26.7	<0.0001
Year x Mixture	18	139876	433	1.5	0.09760
Residuals	120	2722500	188		

df = degrees of freedom; SS = sum of squares; MS = mean square; *F* value = ratio of two variances; *P* value = probability value.

**Table 4:** ANOVA for effects of year, mixture and their interaction on the relative mixing effect on yield cultivated in seven mixtures of three wheat varieties in different ratios during three consecutive years.

	df	SS	MS	*F* value	*P* value
Without fungicide					
Year	2	2256	1128	3.2	0.042
Mixture	6	1631	272	0.8	0.578
Year x Mixture	12	7787	649	1.9	0.047
Residuals	84	28800	343		

With fungicide					
Year	2	1154	577	2.0	0.139
Mixture	6	2827	471	1.7	0.143
Year x Mixture	12	1920	160	0.6	0.867
Residuals	84	23957	285		

df = degrees of freedom; SS = sum of squares; MS = mean square; *F* value = ratio of two variances; *P* value = probability value.

#### 3.1.2 Yield with fungicide

With fungicide application, yields were on average 2587 kg ha^−1^ and 23% higher than in the untreated plots that averaged of 2108 kg ha^−1^ across years. The yield in Year 3 was 3207 kg ha^−1^ on average across mixtures, also significantly higher than in Year 1 with 2396 kg ha^−1^ and Year 2 with 2156 kg ha^−1^. No significant interaction between year and mixture was observed. As in the without fungicide treatment, sole BARI Gom 33 yield was highest at 3339 kg ha^−1^, followed by its mixtures with BARI Gom 30 at 3240 kg ha^−1^ (BARI Gom 30(33) + BARI Gom 33(67)) and 3108 kg ha^−1^ (BARI Gom 30(67) + BARI Gom 33(33)). Neither mixture differed significantly from sole BARI Gom 33 yield.

#### 3.1.3 Relative mixing effect

**Fig. 4:**
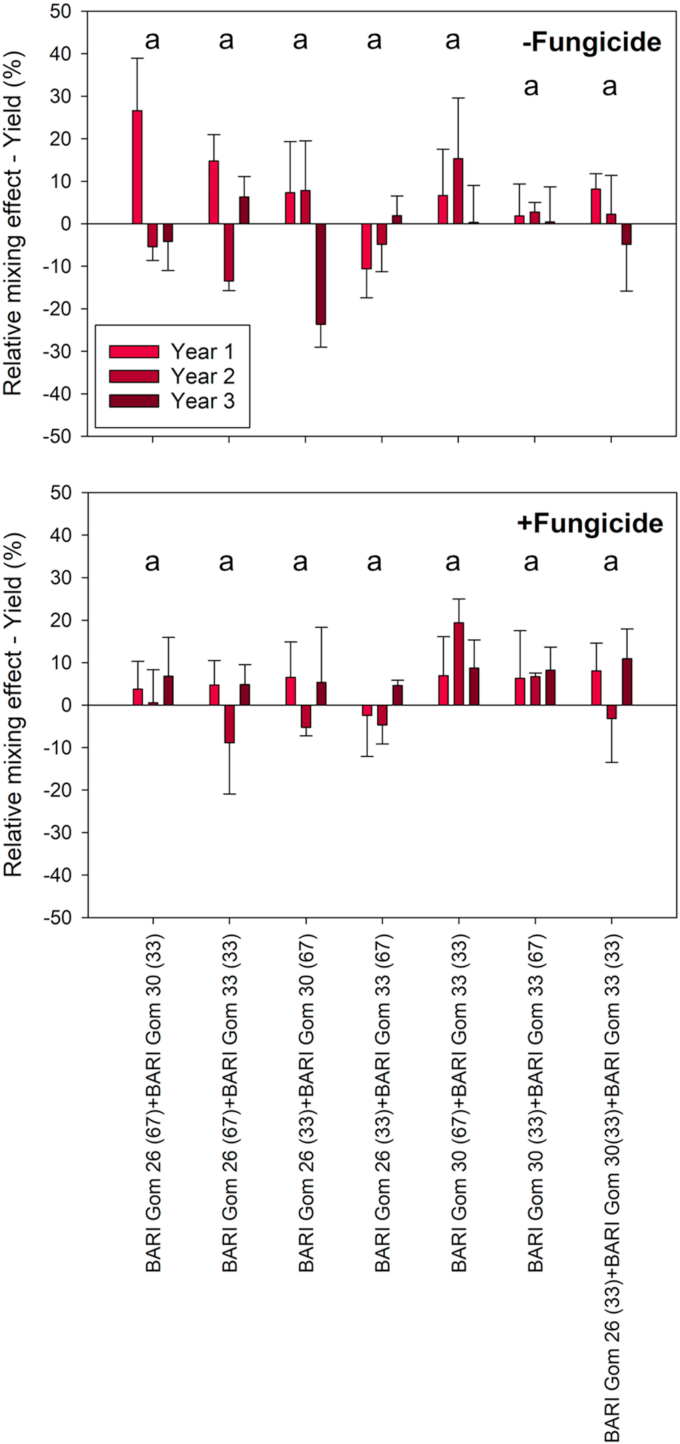
Relative mixing effects on yield of seven variety mixtures of different ratios either with or without fungicide application. The experiment was conducted in three consecutive years, with the third year being the only significant difference observed. Mean values with standard errors are presented. Letters that are not the same indicates statistical differences.

To compare the effect of mixing varieties on grain yield compared to the pure stands, we calculated the relative mixing effect. Without fungicide application, the relative mixing effect ranged from +27% for BARI Gom 26(67) + BARI Gom 30(33) in Year 1 to −24% for BARI Gom 26(33) + BARI Gom 30(67) in Year 3, which was the only statistically significant difference between treatments (Fig. 4). Across all treatments, the relative mixing effect on yield was +2% when no fungicide was applied. When fungicide was applied, the relative mixing effect was +1% across all treatments. With fungicide, no significant effect of year or mixture was found on the relative mixing effect on yield (Table 5).

**Table 5:** ANOVA for the analysis of the effects of year, mixture and their interaction on disease-inflicted yield loss of three wheat varieties cultivated in pure variety stands and seven mixtures of different ratios during three consecutive years without the application of fungicide.

	df	SS	MS	*F* value	*P* value
Year	2	17.9	9.0	9.7	<0.0001
Mixture	9	259.7	28.9	31.1	<0.0001
Year x Mixture	18	20.4	1.1	1.2	0.2539
Residuals	120	111.3	0.9		

df = degrees of freedom; SS = sum of squares; MS = mean square; *F* value = ratio of two variances; *P* value = probability value.

### 3.2 Disease related parameters

#### 3.2.1 Disease inflicted yield loss

**Fig. 5:**
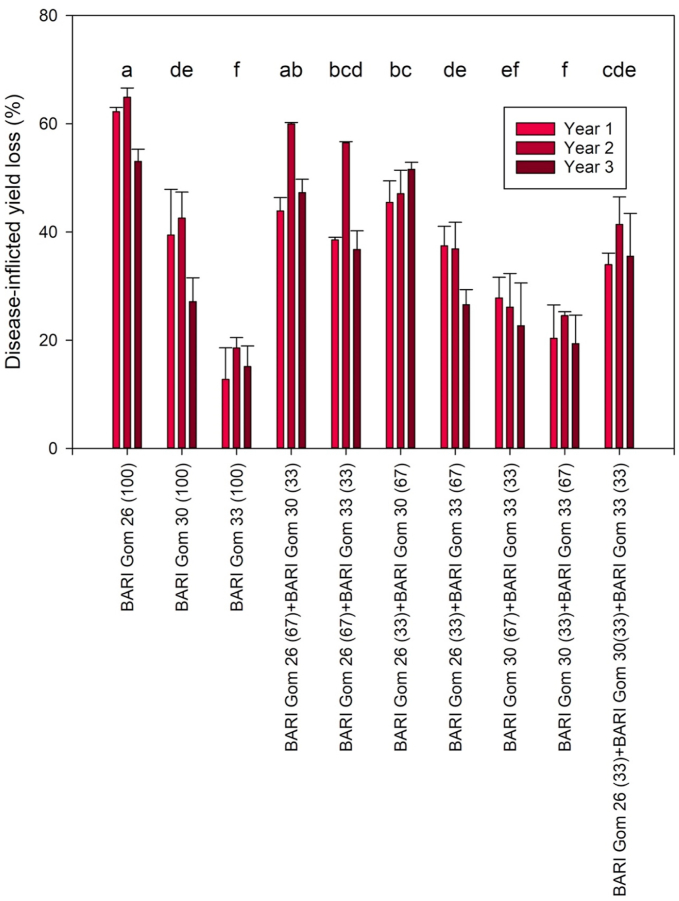
Disease-inflicted yield loss percent of three wheat varieties cultivated in pure variety stands and seven mixtures of different ratios during three consecutive years. Mean values of five replications with standard errors are presented. Different letters indicate significant differences between mixtures at *P* ≤ 0.05. Letters that are not the same indicates statistical differences.

Wheat blast and BpLB disease often appeared in the same plots and/or plants in our experiment. Examining disease-inflicted yield losses, significant differences (*P* < 0.001) were found for Year and Mixture, but not their interaction (Table 5). In Year 2, disease-inflicted yield loss was significantly higher (42 %) than in Year 1 and 3 (36% and 34 %, respectively, on average across mixtures). Without fungicide, the highest disease-inflicted yield loss was found for sole BARI Gom 26 with a 60% average across loss years, followed by its mixtures with a 50% loss and a 48% loss observed BARI Gom 26(67) + BARI Gom 30(33), and BARI Gom 26(33) + BARI Gom 30(67), respectively. Disease-inflicted yield loss without fungicide was lowest for sole BARI Gom 33 at 15%, and mixtures of BARI Gom 33 and BARI Gom 30 were not significantly different from sole BARI Gom 33 in terms of disease-inflicted yield loss (Fig. 5).

#### 3.2.2 Disease incidence, severity and index

**Fig. 6:**
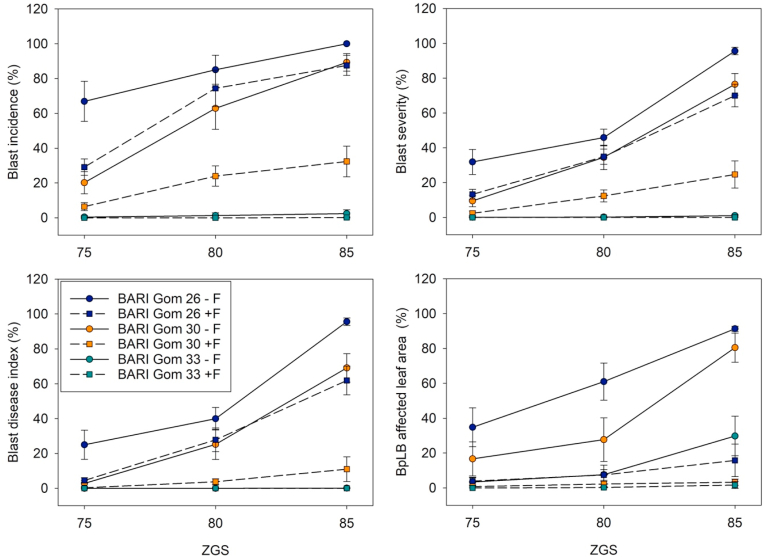
Disease-related parameters (i.e. blast incidence, blast severity, blast disease index, *Bipolaris* leaf blight (BpLB) affected area of the flag leaf) of pure stands of three wheat varieties assessed at Zadok growth stages (ZGS) 75, 80 and 85. Plants were cultivated with (+F) and without fungicide application (-F) during three consecutive years. Presented values are mean values across three seasons (years) and five replications with their corresponding standard errors.

BARI Gom 26 was most affected by both wheat blast and BpLB (Fig. 6). While without fungicide, blast incidence in this variety increased from 77% at 75 ZGS to 100% at 85 ZGS, severity increased from 32% to 96% during the same period. Application of fungicide resulted in substantially lower blast incidence and severity of 29% and 13 %, respectively, in BARI Gom 26 at 75 ZGS, but until 85 ZGS, incidence and severity increased to 88% and 70%, respectively. In contrast, BpLB affected leaf area in BARI Gom 26 without fungicide reached 91% at 85 ZGS, but with fungicide application, it was only 16% at 85 ZGS.

In terms of blast incidence, blast severity, and disease index, BARI Gom 30 without fungicide showed a similar response to BARI Gom 26 after fungicide application. However, fungicide application resulted in drastically lower blast levels in BARI Gom 30, with a disease index of only 11% at 85 ZGS. BpLB affected leaf area was also much lower with fungicide than without, at 3% and 81%, respectively. BARI Gom 33 conversely was barely affected by blast. Even without fungicide, blast incidence at 85 ZGS was 2% in BARI Gom 33, while severity and disease index were 1% and 0%, respectively. With fungicide, all blast-related parameters were 0% at 85 ZGS in BARI Gom 33. BpLB affected leaf area was also lowest in BARI Gom 33, with a maximum of 30% and 2% without and with fungicide, respectively.

**Fig. 7:**
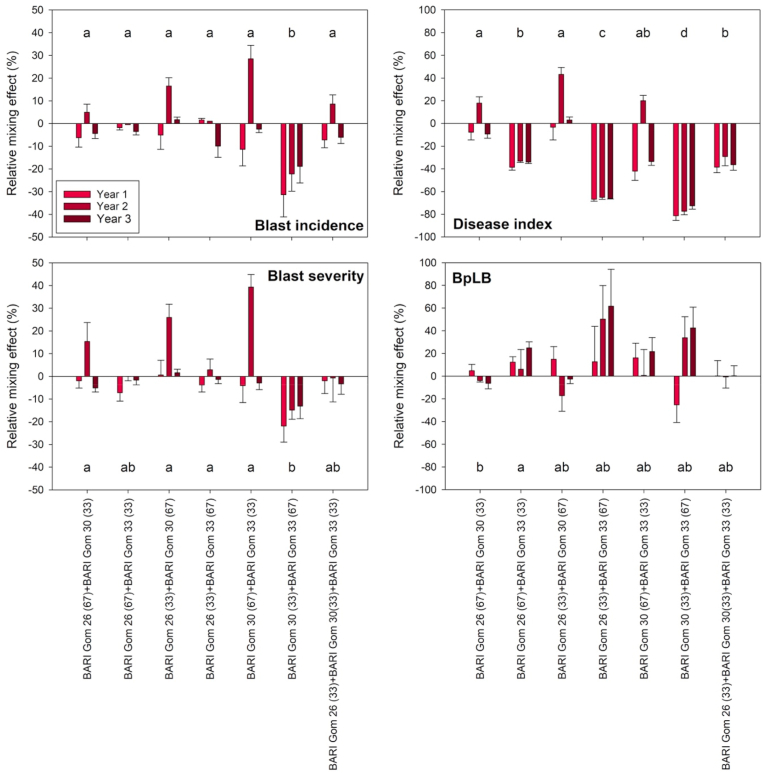
Relative mixing effects of disease-related parameters (i.e. blast incidence, blast severity, blast disease index, *Bipolaris* leaf blight (BpLB) affected area of the flag leaf) of seven variety mixtures of different ratios assessed at Zadok growth stages (ZGS) 85. Plants were cultivated without fungicide application (-F) during three consecutive years. Mean values of five replications with standard errors are presented. Different letters indicate significant differences between mixtures at *P* ≤ 0.05. Letters that are not the same indicates statistical differences.

Without fungicide, a relative mixing effect of −25% on blast incidence was observed for the BARI Gom 30(33) + BARI Gom 33(67) mixture on average across years, which was significantly different from the other mixtures (Fig. 7). A significant and beneficial effect of this mixture was also observed on blast severity, with a relative mixing effect of −1% at 85 ZGS. Considering the disease index, the relative mixing effect was lowest for BARI Gom 30(33) + BARI Gom 33(67) at −77%, but a relative reduction in disease index was also observed for other mixtures containing BARI Gom 33, with a relative mixing effect of −66% for BARI Gom 26(33) + BARI Gom 33(67) and −35% for both BARI Gom 26(67) + BARI Gom 33(33) and BARI Gom 26(33) + BARI Gom 30(34) + BARI Gom 33(33). However, mixing also resulted in a slightly higher proportion of BpLB affected leaf area, with the relative mixing effect averaging between −3% for BARI Gom 26(33) + BARI Gom 30(67) and +31% for BARI Gom 26(33) + BARI Gom 33(67) across years.

#### 3.2.3 Relationships between disease parameters and yield

A consistent and highly significant (*P* < 0.001) negative correlation was found between yield and blast incidence, blast severity, and disease index at 75, 80, and 85 ZGS (Table 6). In contrast, the negative correlation between yield and BpLB affected leaf area was statistically significant (*P* < 0.001) only when BpLB was determined at 85 ZGS. Compared to yield, Pearson correlation coefficients showed an even closer negative correlation between TGW and all disease-related parameters determined at all ZGS. However, of the disease-related parameters, TGW correlated more closely with blast-related parameters than with BpLB affected leaf area. Positive correlation coefficients were found for the relationship between lodging and blast-related parameters but were significant (*P* < 0.05) only in four of nine cases.

**Table 6:** Pearson correlation coefficients between mean values of disease-related wheat blast and *Bipolaris* leaf blight (BpLB) parameters across varieties measured during three Zadok's Growth Stages (75, 80, 85 ZGS) and yield, thousand grain weight (TGW), and lodging of three wheat varieties cultivated in pure variety stands and seven mixtures with different ratios either with or without fungicide application during three consecutive years (*N* = 60).

ZGS		Yield		TGW		Lodging	
75	Blast incidence	−0.72	***	−0.75	***	0.35	**
	Blast severity	−0.76	***	−0.78	***	0.43	**
	Blast disease index	−0.6	***	−0.65	***	0.34	**
	BpLB affected leaf area	−0.17		−0.29	*	−0.03	
80	Blast incidence	−0.80	***	−0.84	***	0.33	*
	Blast severity	−0.74	***	−0.78	***	0.21	
	Blast disease index	−0.71	***	−0.77	***	0.22	
	BpLB affected leaf area	−0.22		−0.37	**	−0.04	
85	Blast incidence	−0.72	***	−0.82	***	0.23	
	Blast severity	−0.70	***	−0.79	***	0.20	
	Blast disease index	−0.68	***	−0.78	***	0.17	
	BpLB affected leaf area	−0.44	***	−0.53	***	0.11	

*, **, ***Indicates significance at *P* < 0.05,<0.01., and 0.001, respectively. *P* indicates the probability value.

**Fig. 8:**
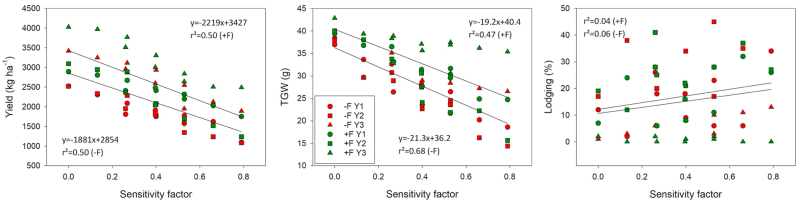
Yield, thousand grain weight (TGW) and percentage of lodged plants, of three wheat varieties cultivated in pure variety stands and seven mixtures of different ratios regressed against the disease sensitivity factor of the variety and the respective mixture, respectively. Wheat was cultivated during three consecutive years either with (+F) or without fungicide application (-F). Values are means of five replications. Regression lines refer to different fungicide treatments, i.e. red: -F; green: +F.

Across all years and fungicide treatments, the disease index measures at 85 ZGS in the pure stands of BARI Gom 26, BARI Gom 30 and BARI Gom 33 were 79%, 40%, and 0%, respectively. These values were used as the baseline for the susceptibility of the respective variety and the sensitivity of the mixtures was calculated according to the proportion of the variety in the mixture. Both with and without fungicide, yield, and TGW negatively and highly significantly correlated with susceptibility, while no significant correlation was found between lodging and sensitivity factor (Fig. 8). Under high blast pressure, a 0.1 increase in the sensitivity factor of the cultivated mixture resulted in a yield loss of about 220 kg ha^−1^ after application of fungicide, while without fungicide application, when yield levels were generally lower, the yield loss was about 190 kg ha^−1^ for each 0.1 increase in sensitivity factor. While variation in TGW was relatively large between years, on average across all years and fungicide treatments, TGW decreased by about 2 g for each 0.1 increase in sensitivity factor of the mixture.

## 4 Discussion and conclusions

Research interest in wheat blast disease has been increasing since the unprecedented 2016 and 2018 outbreaks Bangladesh and Zambia for the first time outside of South America (Malaker et al., 2016; Pequeno et al., 2024). In our study, wheat was severely affected by fungal diseases, though to differing degrees, in each of the three years of the experiment. However, the effects on yield differed between wheat blast as the primary disease of interest, and BpLB as a coincidental disease. Yield with and without fungicide and disease-inflicted yield losses of mixtures of BARI Gom 30 and BARI Gom 33 were not statistically different from sole BARI Gom 33, regardless of the respective proportions of the two varieties in the mixture. Since seeds of BARI Gom 33 are not yet sufficiently available to farmers in Bangladesh in sufficient quantities, mixing with BARI Gom 30 seeds appears to be a viable option to still achieve good yields even under high blast pressure. However, the lower the proportion of BARI Gom 33 and the more susceptible the other component varieties in a given seed mixture, the higher risks of yield loss become. When the sensitivity of the varieties and their mixtures to blast was scaled using a 0 to 1 disease index, yield decreased by about 200 kg ha^−1^ and TGW by about 2 g with each 0.1 increase in the sensitivity factor. It should be noted, however, that these values apply only to a very high blast pressure situation and that lower values are likely at lower disease pressure.

Infestations of BpLB were well controlled by fungicide application, with only BARI Gom 26 showing weak symptoms after application. Since the yields of BARI Gom 26 and BARI Gom 30 still remained far below their reported yield potential of more than 5 t ha^−1^ (Mian, 2021), even after fungicide application, we hypothesize that BpLB had relatively little effect on yield, while the large yield gap was mainly due to blast. Furthermore, the much stronger correlation between blast and yield than between BpLB and yield supports the hypothesis of a stronger influence of blast compared to BpLB. However, challenges with fully controlling one disease but permitting another under field conditions unfortunately did not allow for accurate differentiation between BpLB and blast in terms of yield loss. Siddique et al. (2006) reported yield losses associated with BpLB ranging from 2% to 22%, but also noted a downward trend in disease severity and disease-related grain yield reductions in newer wheat varieties. Further progress has been made in terms of varietal resistance and management of BpLB (Chowdhury et al., 2013) and given the severity of wheat blast outbreak initially reported (Malaker et al., 2016), BpLB has become less of a focus in wheat pathology research in Bangladesh.

Without fungicide, disease-inflicted yield loss was as high as 65% but varied among years and especially among varieties and variety mixtures. de Campos Dianese et al. (2021) found a very wide range of varietal responses when comparing different varieties under high blast pressure. They reported blast-related yield losses of 87%–98% at 100% blast incidence, while other varieties with resistant traits showed very little yield loss with either no or minor symptoms, or with pronounced symptoms indicating blast tolerance. However, close correlation between blast symptoms and disease-inflicted yield losses is common (de Campos Dianese et al., 2021; de Coelho et al., 2016), indicating that blast tolerance tends to be the exception, while resistance to blast can and should be used for more optimal disease management.

Fungicide application resulted in 23% higher yields on average, but yields of the blast susceptible BARI Gom 26 and the moderately blast-tolerant BARI Gom 30 were still 45% and 22% lower, respectively, than those of blast resistant BARI Gom 33. Mian (2021) reported the same yield potential for all three of these varieties; consequently, low fungicide efficacy against blast can be inferred from our data. In general, fungicide efficacy against wheat blast is considered low (Ceresini et al., 2018; Zhang et al., 2022). At relatively low blast pressure, blast symptoms in the susceptible BARI Gom 26 have been reasonably controlled with Nativo75 (Roy et al., 2021). When comparing a large number of trials, the efficacy of the combination of trifloxystrobin and tebuconazole used in this experiment was on average 52% and was in the same range as most other fungicides studied (Ascari et al., 2021). However, the same study also described generally lower efficacy of fungicides against blast in the humid tropics and sub-tropics, which exacerbates the problem for Bangladesh.

Given the high yield losses and low efficacy of fungicides against blast, our study underscores that the use of resistant varieties is of paramount importance. This result is relevant in Bangladesh but also likely to hold significance in many wheat growing areas challenged by this disease in other countries. Comparison of the three varieties used in this experiment showed the superior performance of BARI Gom 33. However, despite showing very limited visible blast symptoms, disease-inflicted yield loss of BARI Gom 33 ranged from 13% to 19% over the three years when compared to fungicide application. Although BARI Gom 33 also showed the greatest resistance to BpLB among the three varieties tested, without fungicide, its BpLB affected leaf area was also 29.8% on average. Our data therefore suggest that in the case of blast-resistant BARI Gom 33, a significant proportion of the disease-inflicted yield loss is likely to have been caused by BpLB, underscoring the importance of integrated disease management as these diseases can be coincidental.

In our experiment, limited negative effect of mixing varieties on yield was found, confirming studies summarized by Vestergaard and Jørgensen (2024). Very small to no effects of mixtures on wheat yield have been reported under non-stress conditions (Dai et al., 2012; Lazzaro et al., 2018), while slightly positive effects of variety mixtures have been measured average across multiple environments (Gallandt et al., 2001) and in a meta-analysis (Kiᴂ;r et al., 2009). However, a more recent meta-analysis showed a more pronounced yield advantage of mixtures under high disease pressure and also for mixtures that were heterogeneous in terms of disease resistance (Borg et al., 2018). In our case, while both high disease pressure and heterogeneity were present and yet they did not result in a yield advantage. This could have been due to the effect of frequent artificial inoculation by sprinklers. Under natural field conditions, mixtures of varieties can delay the spread of pathogens as resistant plants and/or plants with different canopy architecture can act as barriers to the spread of disease as spores are transported unevenly across the crop (Kiᴂ;r et al., 2009). In our study, however, the aerosols produced by spraying inoculum were uniform and frequent, with misting used to help facilitate movement of spores from spreader rows evenly across plots, which could have affected the efficacy of varietal mixing in disease control. Further experimentation with mixtures – which had no significantly negative effect on yield, and could provide an option for farmers lacking sufficient quantities of resistant varieties like BARI Gom 33 – under natural disease pressure and under conditions found in farmer's fields is therefore warranted.

On the other hand, the effects of variety mixtures were evident in terms of disease symptoms. While mixing varieties tended to increase BpLB symptoms, blast incidence and disease index were lower on average compared to sole variety stands. Differences were also observed among mixtures, with the BARI Gom 30(33) + BARI Gom 33(67) mixture showing a significant relative mixing effect with lower values for blast incidence and disease index compared to all other mixtures and for blast severity compared to most other mixtures. This suggests that mixtures could potentially help to suppress the generation of inoculum that could spread to other fields, though as suggested above, further experimentation under on-farm field conditions would be beneficial. Disease reduction in mixtures has been explained by different survival rates and/or tiller production of mixture components in mixtures of resistant and susceptible cultivars (Finckh and Mundt 1992). In our case, neither pathogen growth nor the individual variety yield components were studied, but since inoculation with blast spores started at the maximum tiller stage, higher survival rate and/or tiller production associated with blast resistance is unlikely. Another explanation for the decline in disease in mixtures is the epidemiological effect of linking lower spatial density of susceptible plants to limited spore dispersal, as spore density decreases exponentially along a gradient from the source (Wolfe, 1985). Increased spacing between susceptible plants is thought to be a contributing factor in disease reduction in variety mixtures (Finckh et al., 2000). This effect should be greatest in heterogeneous mixtures, as resistant varieties provide the more susceptible protection (Borg et al., 2018). The most commonly recommended row-to-row spacing for wheat in Bangladesh is 20 cm (BARI 2014). In the current study, however, we did not vary row-to-row spacing, although in practice farmers may modify spacing which could affect the aerodynamics of spore movement and infection within the canopy (Gagan et al., 2021). Further research could therefore investigate the implications of different spacings with cultivar mixtures and potential trade-offs with yield component formation in wheat. The effect of the mixture on blast symptoms in our experiment was best when the resistant BARI Gom 33 dominated in the mixture. However, the most favorable relative mixing effect was obtained when BARI Gom 33 was mixed with the moderately resistant BARI Gom 30 and not, as might have been expected, with the susceptible BARI Gom 26.

Although choice of variety is probably the most important tool to mitigate blast-related yield reductions, the evolution of diseases to overcome resistance in cultivars will remain a concern and other management measures should also be used in the context of comprehensive integrated disease management. This is because disease-inflicted yield losses were observed even in the highly resistant BARI Gom 33. While foliar application of fungicides is recommended in many countries to control wheat blast dispersed aerially (Pequeno et al., 2024), the disease is also seed-borne and seed treatment can consequently help prevent symptoms during early growth stages (Sadat and Choi, 2017) or even eliminate the seed-borne infection (Islam et al., 2019). In addition to chemical control, changing sowing dates to reduce exposure of the crop to weather conditions conducive to disease has also been recommended in both literature review and continental-scale modeling studies (Kohli et al., 2011; Montes et al., 2022). In Brazil, late sowing resulted in lower blast infection and it has been argued that mean minimum temperatures below 14 °C and relative humidity less than 60% contribute to a reduction in disease incidence in wheat (de Coelho et al., 2016), while Fernandes et al. (2017) suggested that relative humidity <93% limited wheat blast inoculation potential. Since high temperatures towards the end of the wheat cropping season are common in Bangladesh (Mian et al., 2019), earlier sowing, which tends to have yield advantages (Krupnik et al., 2015a, Krupnik et al., 2015b) could also help to avoid the critical period that favors the spread of the disease (Montes et al., 2022). Another approach that was imposed by the Government of Bangladesh to avoid the disease was to suspend wheat production in highly blast-prone areas, as discussed by Mottaleb et al. (2019). However, this approach was only implemented in the 2016–17 wheat season following the first blast outbreak in Bangladesh. Given Bangladesh's high dependence on wheat imports and foreign currency reserve concerns, suspending wheat production to control diseases is unlikely to be a viable long-term option. In conclusion, our results suggest that to sustain wheat production in Bangladesh, farmers can make use of the resistant variety BARI Gom 33 in pure stands or in mixtures of moderately susceptible varieties to maintain yields on-par with sole stands of BARI Gom 33, although prudent application of fungicides may also be considered under very high disease pressure conditions. Similar research on the performance of varietal mixtures and under high disease pressure conditions can and should however be repeated in other countries in which wheat blast is endemic to develop context-specific, tailored recommendations that can be applied by farmers as part of integrated disease management strategies.

## CRediT authorship contribution statement

**Timothy J. Krupnik:** Writing – review & editing, Writing – original draft, Methodology, Funding acquisition, Formal analysis, Conceptualization. **Md. Harun-Or-Rashid:** Writing – review & editing, Writing – original draft, Methodology, Investigation, Data curation. **Dinabandhu Pandit:** Methodology, Investigation, Conceptualization. **Rabiul Islam:** Validation, Investigation. **Md. Khaled Hossain:** Writing – review & editing, Data curation. **José Mauricio Cunha Fernandes:** Writing – review & editing, Validation. **Krishna Kanta Roy:** Writing – review & editing, Validation. **Muhammad Rezaul Kabir:** Writing – review & editing, Validation. **Sabine Stuerz:** Writing – review & editing, Writing – original draft, Visualization, Methodology, Formal analysis. **Pawan Kumar Singh:** Writing – review & editing, Validation. **Golam Faruq:** Writing – review & editing, Validation.

## Declaration of competing interest

The authors declare the following financial interests/personal relationships which may be considered as potential competing interests:

Timothy J. Krupnik reports financial support was provided by 10.13039/100000200USAID, 10.13039/501100000974ACIAR, the 10.13039/100000865Bill and Melinda Gates Foundation, and 10.13039/501100015815CGIAR. All other authors declare that they have no known competing financial interests or personal relationships that could have appeared to influence the work reported in this paper.

## Data Availability

Data will be made available on request.
